# Association of Elevated Plasma Total Homocysteine With Dementia With Lewy Bodies: A Case-Control Study

**DOI:** 10.3389/fnagi.2021.724990

**Published:** 2021-10-15

**Authors:** Guili Zhang, Shuai Liu, Zhichao Chen, Zhihong Shi, Wenzheng Hu, Lingyun Ma, Xiaodan Wang, Xudong Li, Yong Ji

**Affiliations:** ^1^Department of Neurology, Beijing Tiantan Hospital, Capital Medical University, Beijing, China; ^2^China National Clinical Research Center for Neurological Diseases, Beijing, China; ^3^Tianjin Key Laboratory of Cerebrovascular and of Neurodegenerative Diseases, Tianjin, China; ^4^Department of Neurology, Tianjin Dementia Institute, Tianjin Huanhu Hospital, Tianjin, China

**Keywords:** Alzheimer’s disease, dementia with Lewy bodies, homocysteine, folate, vitamin B12

## Abstract

**Background**: Elevated plasma total homocysteine (tHcy) level, a known risk factor for vascular disease, is reported to be an independent risk factor for cognitive impairment and Alzheimer’s disease (AD) in most studies. tHcy may also be associated with dementia with Lewy bodies (DLB).

**Objective**: To investigate the association between plasma tHcy levels and DLB or AD.

**Methods**: This is a case-control study including 132 DLB patients, 264 AD patients, and 295 age-matched healthy controls. We used multivariate logistic regression model to analyze the data with adjustments for confounding variables.

**Results**: The highest tHcy tertile (>13.9 μmol/L) was significantly independently associated with DLB [adjusted odds ratio (OR): 4.65, 95% confidence interval (CI): 1.95–11.10, *P* = 0.001] and AD (adjusted OR: 1.82, 95% CI: 1.02–3.23, *P* = 0.041) compared to the lowest tertile (<10.7 μmol/L). The cumulative frequency plots showed a shift in the distribution of the tHcy concentrations to higher values in patients with DLB compared to AD. The mean tHcy levels were stable and not altered by the duration of cognitive impairment prior to the collection of blood samples from DLB patients.

**Conclusion**: Elevated plasma tHcy levels were independently associated with DLB, and the association was stronger for DLB than for AD. The lack of a relationship between tHcy levels and symptom duration may refute these observed associations being a consequence of DLB, and future longitudinal studies will be required to confirm whether tHcy plays a causative role in DLB.

## Introduction

Dementia with Lewy bodies (DLB) is the second most common neurodegenerative cause of dementia in older people following Alzheimer’s disease (AD; Walker et al., 2015). DLB is characterized by cognitive impairment and core clinical features including fluctuating cognition, visual hallucinations (VH), Parkinsonism, and rapid eye movement (REM) sleep behavior disorder (RBD; McKeith et al., 2017; Mueller et al., 2017). According to previous epidemiological studies, the prevalence estimates of DLB range from 0% to 30.5% of all patients with dementia (Yue et al., 2016; Galvin and Bras, 2018). Higher mortality and greater caregiver burdens are also found in DLB compared to AD due to a combination of cognitive impairment and core clinical symptoms such as pronounced hallucinations, extrapyramidal symptoms, autonomic dysfunction, and sleeping difficulties (Galvin et al., 2010; Liu et al., 2017a,b, 2018). Therefore, it is important to identify the treatable risk factors for cognitive dysfunction and the core symptoms of DLB to reduce the prevalence and the caregiver burdens of this disease.

B vitamins including vitamin B12 and folate are essential factors involved in nucleotide synthesis, one-carbon metabolism, and DNA-methylation; they are also necessary for the well-being and normal function of the brain and have been linked to cognitive impairment, AD, and dementia, though results have been inconclusive thus far (Clarke et al., 1998; Ravaglia et al., 2005; Gillette Guyonnet et al., 2007; Pusceddu et al., 2017; Rabensteiner et al., 2020; Hoffmann et al., 2021). Homocysteine, the cytotoxic product of the methionine cycle, is a sensitive marker of folate and vitamin B12 status and may have a direct toxic effect on both blood vessels and neurons (Welch and Loscalzo, 1998; Ho et al., 2002; Refsum et al., 2004; Pusceddu et al., 2017). Furthermore, elevated total homocysteine (tHcy) levels are not only a risk factor for vascular diseases but also for cognitive impairment and AD (Clarke et al., 1991; Seshadri et al., 2002; Ravaglia et al., 2005). Elevated levels of tHcy are were also related to dopaminergic neuron loss and impaired cognitive function in Parkinson’s disease (PD; Bhattacharjee et al., 2016; Sleeman et al., 2019). Neuropathological findings showed that DLB and PD shared the same pathological hallmarks such as abnormal α-synuclein deposition (Jellinger and Korczyn, 2018). Notably, more recent studies showed the co-existence of cerebrovascular pathology and Aβ accumulation in patients with DLB, implying DLB is featured by the co-occurrence of Lewy/α-synuclein, cerebrovascular, and AD-related pathologies (De Reuck et al., 2013; Galvin and Bras, 2018). On the basis of these studies, it has been hypothesized that elevated plasma tHcy levels, a risk for vascular disease and AD, may play a role in DLB. To date, the associations of differing levels of plasma tHcy and serum folate and vitamin B12 in patients with DLB remain unreported.

In the present study, we used a case-control design to evaluate the associations of tHcy, folate, and vitamin B12 levels with DLB and AD patients.

## Materials and Methods

### Study Design and Subjects

**Figure 1:**
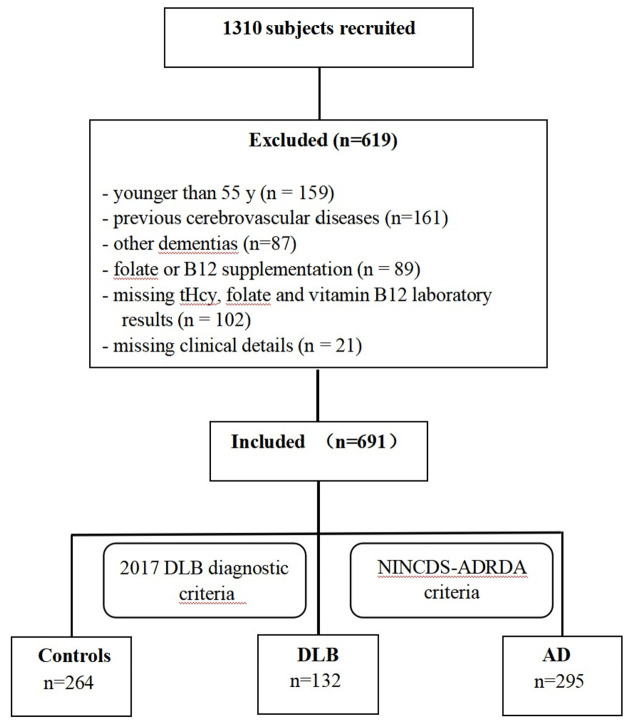
Flowchart of study subjects selection.

This case-control study was designed to examine the association between plasma tHcy, serum folate, and vitamin B12 levels and Chinese patients with DLB and AD. Between 2017 and 2020, the recruitment, selection, and classification of subjects were performed according to a flow chart shown in Figure 1. A total of 1,310 participants (>55 years of age) were consecutively recruited from the memory clinic at Beijing Tiantan Hospital in China, which is a center of the China Lewy Body Disease Collaborative Alliance. Subjects younger than 55 years old (*n* = 159), individuals whose magnetic resonance exam showed cerebrovascular lesions (*n* = 161), or dementia patients with conditions other than AD or DLB (*n* = 87) were excluded. Additionally, 212 individuals were excluded for the following reasons: their plasma tHcy, serum folate, and vitamin B12 concentrations were unavailable (*n* = 102); they were suffering from malnutrition and vitamin B12 deficiency; they were treated with folate or vitamin B12 in the last 3 months (*n* = 89); or their clinical details were absent (*n* = 21). In total, the remaining 691 subjects-including 132 DLB, 295 AD patients, and 264 healthy controls-constituted our study sample and were examined by a committee comprised of at least two neurologists and one neuropsychologist. Dementia was diagnosed based on the clinical criteria of the Diagnostic and Statistical Manual of Mental Disorders, 4th edition (DSM-IV; American Psychiatric Association, 1994). Patients with probable DLB were clinically diagnosed according to the fourth consensus report of the DLB Consortium by McKeith et al. (2017). For these patients, dementia had to start at least 1 year before the onset of the extrapyramidal syndrome (otherwise, they would be diagnosed with PD dementia) and initially present with at least two core clinical features of DLB (visual hallucinations, Parkinsonism, fluctuating cognition, and/or RBD) were shown at the first presentation at our memory clinic. Furthermore, ^18^F-FDG PET scans of patients showing biomarkers such as occipital hypometabolism and/or the cingulate island sign warranted a probable DLB diagnosis. Patients with probable AD (*n* = 295) who were clinically diagnosed according to the National Institute of Neurological and Communicative Disorders and Stroke and the Alzheimer’s Disease and Related Disorders Association (NINCDS-ADRDA) criteria (McKhann et al., 1984), and ^11^C-PIB PET scans to assess Aβ deposition were performed. Healthy controls were considered free of cognitive impairments with Mini-Mental State Examination (MMSE) scores of >26. This study was designed and conducted in accordance with the Declaration of Helsinki, and written informed consent was obtained from all participants.

### Clinical and Neuropsychological Assessment

All subjects underwent a standardized diagnostic workup that included a semistructured medical history interview, collection of an informant-based history, neurological and physical examinations, a neuropsychological assessment, a brain MRI, and a standard laboratory test. The MMSE was used to assess the severity of cognitive impairment. Overall cognitive function was evaluated using a clinical dementia rating (CDR) and CDR global scores (range 0–3) were recorded. Neuropsychiatric symptoms (NPS) were assessed with the 12-item Neuropsychiatric Inventory (NPI) using the information provided by the caregiver.

### DLB Core Clinical Features Assessment

According to Marleen van de Beek et al. study in 2019 (the presence of core clinical features (fluctuating cognition, Pparkinsonism, visual hallucinations, and RBD) was assessed and obtained from the standardized clinical diagnostic workup (described previously; van de Beek et al., 2020). Hallucinations were systematically assessed and scored by NPI (hallucinations present: ≥1). Parkinsonism was rated as present when the neurological exam showed extrapyramidal signs (tremor, bradykinesia, and/or rigidity). RBD was rated as present according to caregivers’ reports that the patients seemed to “act out” their dreams and were moving extensively during sleep. Fluctuating cognitions including memory, attention, executive functions, language, and visuospatial functions were rated positively according to the patients’ or caregiver’s reports that the patients’ cognitive status fluctuated during the day and over weeks.

### Laboratory Tests

Participants’ blood samples were obtained following an overnight fast of 12–14 h. The samples were drawn by venipuncture into 5-ml plain evacuated tubes and then centrifuged at 2,000 *g* for 10 min. All specimens were collected in plastic vacuum tubes containing EDTA and analyzed within 1 h or stored at −80°C until use. Plasma tHcy, serum folate, and serum vitamin B12 were determined by electrochemiluminescence immunoassays (ECLIA) from Roche Diagnostics that were run on a COBAS 8000 e 602 analyzer (Roche Diagnostics, Switzerland).

### Statistical Analysis

Continuous variables are expressed as the mean and standard deviation while categorical variables are expressed as proportions. To assess differences among the healthy control, DLB, and AD groups, an analysis of variance (ANOVA) followed by Dunnett’s test was used for continuous variables, and a chi-squared test followed by a Bonferroni-corrected pairwise comparison test was used for categorical variables.Multivariate logistic regression analysis was used to estimate the associations of plasma tHcy, serum folate, and serum vitamin B12 concentrations with DLB and AD. Reference tertiles were the top tertiles for folate and vitamin B12 and the bottom tertile for plasma tHcy. In the multivariate logistic regression analysis models, folate, vitamin B12, and tHcy together with age, sex, education, smoking status, alcohol intake, type 2 diabetes mellitus (T2DM), coronary artery disease (CAD), and hypertension were entered as covariates. All analyses were conducted in the following four models: model 1: Unadjusted; model 2: Adjusted for age, sex, and education; model 3: Adjusted for model 2 + tHcy, folate; and vitamin B12; and model 4: Adjusted for model 3 + smoking status, alcohol intake, T2DM, CAD and hypertension. Statistical significance was defined as a *P*-value of <0.05. All statistical analyses were performed using SPSS 25.0 (IBM Corp., Armonk, NY, USA).

## Results

### Characteristics of Participants

The clinical and biochemical characteristics of the study subjects are shown in Table 1. The three groups (healthy controls, DLB, and AD) were matched for age, gender, years of education, and alcohol drinking status. The DLB and AD groups had a higher proportion of hypertension, T2DM, CAD, and smokers than the control group (*P* < 0.01). There were no differences in mean MMSE scores between the DLB (15.3 ± 7.2) and AD (14.5 ± 6.1) groups. The DLB patients scored higher on the NPI when compared with the AD patients (*P* < 0.01), which indicated more NPS in the DLB patients. DLB patients also had higher tHcy levels (22.9 ± 16.3 μmol/L; *P* < 0.05) and lower vitamin B12 levels (337.2 ± 210.4 pmol/L; *P* < 0.05) relative to AD patients (18.4 ± 11.6 μmol/L and 397.0 ± 213.9 pmol/L, respectively). In addition, Table 1 shows that fluctuations were the most frequent core clinical feature (81.1%), whereas hallucinations, RBD, and parkinsonism were reported in above half of all 132 DLB patients (74.2%, 63.6%, and 56.8%, respectively).

**Table 1:** Characteristics of Control, DLB, and AD Groups.

Variable	Controls (*n* = 264)	DLB (*n* = 132)	AD (*n* = 295)	*P* value
Clinical variables				
Age, mean (SD), y	72.2 (6.1)	72.7 (7.8)	71.9 (8.6)	0.474
Sex, male, *n* (%)	118 (44.6)	64 (48.5)	145 (48.9)	0.518
Education, mean(SD), y	9.8 (2.9)	9.7 (4.8)	9.5 (4.3)	0.661
Disease duration, mean(SD), m	0	36.3 (23.8)	39.6 (27.4)	0.899
Smokers, *n* (%)	44 (16.6)	41 (31.1)^b^	92 (31.0)^b^	<0.001
Alcohol intake, *n* (%)	38 (14.3)	21 (15.9)	54 (18.2)	0.453
Hypertension, *n* (%)	82 (31.0)	60 (45.5)^b^	134 (45.2)^b^	0.001
T2DM, *n* (%)	18 (6.8)	31 (23.5)^bc^	41 (13.8)^b^	0.004
CAD, *n* (%)	20 (7.5)	22 (16.7)^b^	39 (13.1)^a^	0.017
MMSE sore, mean (SD)	25.4 (1.3)	15.3 (7.2)^b^	14.5 (6.1)^b^	<0.001
NPI total scores	2.6 (2.4)	20.0 (15.4)^bd^	11.7 (13.4)^b^	<0.001
CDR	0	1.8 (0.8)	1.8 (0.8)	0.857
DLB core features, *n* (%)				
Visual hallucination	0	98 (74.2)	13
Parkinsonism	0	75 (56.8)	0	
Cognitive fluctuation	0	101 (81.1)	0
RBD	0	84 (63.6)	0	
Biochemical variables				
Plasma tHcy, μmol/L	13.0 (4.4)	22.9 (16.3)^bc^	18.4 (11.6)^b^	<0.001
Serum Folate, nmol/L	14.3 (8.0)	8.0 (5.9)^b^	8.4 (9.1)^b^	<0.001
Serum Vitamin B12, pmol/L	457.5 (205.6)	337.2 (210.4)^bc^	397.0 (213.9)^b^	<0.001

*Results are shown as *n* (%) for the chi-square or Fisher’s exact tests and as the mean ± SD for ANOVA or Kruskal-Wallis test. DLB, dementia with Lewy bodies; AD, Alzheimer’s disease; RBD, rapid eye movement (REM) sleep behavior disorder; T2DM, type 2 diabetes mellitus; CAD, coronary artery disease; tHcy, total homocysteine; MMSE, Mini-Mental State Examination; NPI, neuropsychiatric inventory; CDR, clinical dementia rating; ANOVA, analysis of variance. Significance was indicated by the following *p* values: a, *P* < 0.05 vs. controls; b, *P* < 0.01 vs. controls; c, *P* < 0.05 DLB vs. AD; d, *P* < 0.01 DLB vs. AD*.

### Association Between Plasma Total Homocysteine, Serum Folate, and Vitamin B12 With DLB and AD

The crude (unadjusted) and adjusted ORs for the DLB and AD groups according to the tertile concentrations of plasma tHcy, serum folate, and serum vitamin B12 calculated using the multivariate logistic regression analyses are shown in Table 2. Increased tHcy levels and reduced folate levels were strongly associated with both DLB and AD patients independent of age, sex, education, and folate, vitamin B12 or tHcy, as well as known or putative vascular risk factors. When the lowest tHcy tertile (<10.7 μmol/L) were used as the reference group, the highest tertile (>13.9 μmol/L) and the median tertile (10.7–13.9 μmol/L) were associated with both DLB [highest tertile crude OR: 10.21, 95% confidence interval (CI), 4.84–21.52; *P* < 0.001; median tertile crude OR: 3.08, 95% CI, 1.38–6.88; *P* = 0.006] and AD (high tertile crude OR: 3.98, 95% CI: 2.65–6.72, *P* < 0.001; median tertile crude OR: 1.87, 95% CI: 1.16–3.00; *P* = 0.01). These associations remained significant with adjustment for confounding variables. After adjustment for age, sex, education, folate concentration, vitamin B12 concentration, and known or putative vascular risk factors including smoking status, alcohol intake, T2DM, CAD, and hypertension in model 4, the ORs of DLB and AD comparing the highest tertile with the lowest tHcy tertile, respectively, were still statistically significant 4.65 (95% CI: 1.95–11.10, *P* = 0.001) and 1.82 (95% CI: 1.02–3.23, *P* = 0.041). Furthermore, DLB and AD patients in the median tHcy tertile had an significant adjusted OR of 3.20 (95% CI: 1.43–7.18, *P* = 0.005) and 1.95 (95% CI: 1.20–3.15, *P* = 0.007), independent of age, sex, and education in model 2, but the ORs of 1.41 (95% CI: 0.59–3.45, *P* = 0.449) and 0.81 (95% CI: 0.46–1.43, *P* = 0.471) for DLB and AD patients were no longer significant after adjustment for folate and vitamin B12 concentrations in model 3. In addition, compared to the highest tertile, the lowest folate tertile was associated with both DLB (crude OR: 10.10, 95% CI: 4.95–20.62; *P* < 0.001) and AD (crude OR: 14.19, 95% CI: 7.89–25.51; *P* < 0.001). Furthermore, the observed association between low folate levels and DLB (adjusted OR: 6.89, 95% CI: 3.08–15.40; *P* < 0.001) or AD (adjusted OR: 12.84, 95% CI: 6.59–25.01; *P* < 0.001) remain significant after adjustment for age, sex, education, vitamin B12 and tHcy concentrations, or common vascular risk factors. However, the lowest serum vitamin B12 levels were mildly related to DLB and AD. The crude OR of DLB and AD in the lowest vitamin B12 tertile is 3.25 (95% CI: 1.89–5.60; *P* < 0.001) and 1.68 (95% CI: 1.12–2.51; *P* = 0.012), respectively. The observed association of the lowest vitamin B12 for DLB patient with an adjusted OR of 1.97 (95% CI: 1.03–3.79, *P* = 0.041) remain significant independent of age, sex, education, or tHcy and folate concentrations. Alternatively, the ORs of AD with the lowest vitamin B12 tertile were significant 1.66 (95% CI: 1.10–2.49, *P* = 0.016) after adjustment for age, sex, and education in model 2. However, this association between the lowest vitamin B12 tertile and both DLB and AD was no longer significant after adjustment for all confounding variables in model 4.

**Table 2:** Mutivariable logstic regression models examining the association between the plasma tHcy and DLB or AD.

	DLB				AD			
Tertiles tHcy, μmol/LI	Model 1	Model 2	Model 3	Model 4	Model 1	Model 2	Model 3	Model 4
I < 10.7	Reference	Reference	Reference	Reference	Reference	Reference	Reference	Reference
II 10.7 –13.9	3.08 (1.38–6.88) ***P* = 0.006**	3.20 (1.43–7.18) ***P* = 0.005**	1.41 (0.59–3.45) *P* = 0.449	1.60 (0.64–4.00) *P* = 0.315	1.87 (1.16–3.00) ***P* = 0.010**	1.95 (1.20–3.15) ***P* = 0.007**	0.81 (0.46–1.43) *P* = 0.471	0.93 (0.52–1.65) *P* = 0.79
1III > 13.9	10.21 (4.84–21.52) ***P* <0.001**	10.73 (5.03–22.88) ***P* <0.001**	4.75 (2.03–11.08) ***P* <0.001**	4.65 (1.95–11.10) ***P* = 0.001**	3.98 (2.65–6.72) ***P* <0.001**	4.22 (2.65–6.72) ***P* <0.001**	1.91 (1.09–3.33) ***P* = 0.023**	1.82 (1.02–3.23) ***P* = 0.041**
**Folate, nmol/L**		
III > 17.4	Reference	Reference	Reference	Reference	Reference	Reference	Reference	Reference
II 9.6–17.4	2.10 (0.94–4.72) *P* = 0.072	2.17 (0.96–4.89) *P* = 0.062	1.61 (0.67–3.84) *P* = 0.287	1.58 (0.66–3.81) *P* = 0.308	3.25 (1.73–6.12) ***P* <0.001**	3.22 (1.70–6.10) ***P* <0.001**	3.35 (1.72–6.52) ***P* <0.001**	3.17 (1.61–6.26) ***P* = 0.001**
I< 9.6	10.10 (4.95–20.62) ***P* <0.001**	11.01 (5.31–22.80) ***P* <0.001**	7.73 (3.50–17.06) ***P* <0.001**	6.89 (3.08–15.40) ***P* <0.001**	14.19 (7.89–25.51) ***P* <0.001**	15.30 (8.41–27.83) ***P* <0.001**	14.86 (7.82–28.25) ***P* <0.001**	12.84 (6.59–25.01) ***P* <0.001**
**Vitamin B12, pmol/L**		
III > 513.3	Reference	Reference	Reference	Reference	Reference	Reference	Reference	Reference
II 337.1–513.3	1.25 (0.68–2.31) *P* = 0.471	1.24 (0.67–2.29) *P* = 0.501	0.82 (0.40–1.67) *P* = 0.582	0.82 (0.40–1.71) *P* = 0.602	0.96 (0.63–1.48) *P* = 0.863	0.94 (0.61–1.45) *P* = 0.791	0.68 (0.41–1.12) *P* = 0.130	0.71 (0.42–1.20) *P* = 0.201
I< 337.1	3.25 (1.89–5.60) ***P* <0.001**	3.21 (1.85–5.55) ***P* <0.001**	1.97 (1.03–3.79) ***P* = 0.041**	1.94 (1.00–3.76) *P* = 0.051	1.68 (1.12–2.51) ***P* = 0.012**	1.66 (1.10–2.49) ***P* = 0.016**	1.00 (0.61–1.65) *P* = 0.992	1.01 (0.61–1.68) *P* = 0.971

*tHcy, total homocysteine; Model 1: unadjusted. Model 2: adjusted for age, sex, and education. Model 3: adjusted for age, sex, education, tHcy, folate, and vitamin B12. Model 4: adjusted for age, sex, education, tHcy, folate, vitamin B12, smoking status, alcohol intake, T2DM, CAD, and hypertension. Bold values indicate statistical significance*.

**Figure 2:**
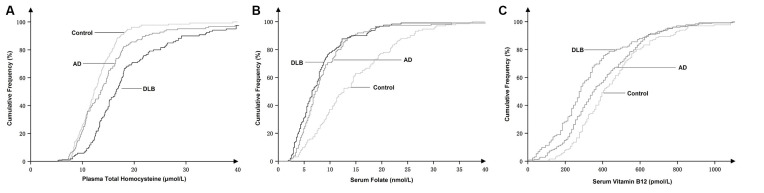
Cumulative frequency distributions of plasma total homocysteine (tHcy) **(A)**, serum folate **(B)**, and serum vitamin B12 concentrations **(C)** in DLB and AD patients and healthy controls.

The cumulative frequency distributions of the tHcy, folate, and vitamin B12 concentrations in the three groups are shown in Figure 2. The cumulative frequency of tHcy, folate, and vitamin B12 grew together in the DLB group and the AD group but appeared distinctly disjoined in the healthy control group. There was a marked shift in the distribution of the tHcy concentrations to higher values and both the folate and vitamin B12 concentrations to lower values in both patients with DLB and AD compared with healthy controls. The cumulative frequency distributions of tHcy in the DLB group appeared markedly separate in the AD group. The cumulative frequency plots showed a shift in the distribution of the tHcy concentrations to higher values in the DLB group compared with the AD group (Figure 2).

### Influence of DLB Duration on Biochemical and Clinical Variables

To investigate whether the prior duration of DLB could explain the observed biochemical changes, the available data of 132 DLB patients were classified by tertiles of cognitive impairment duration (as reported by an informant) before blood samples were taken (Table 3). The severity of cognitive impairment (MMSE score) and neuropsychiatric symptoms (NPI score) were substantially greater in those with a longer duration of illness, but there was no significant trend in the mean plasma tHcy concentration with the increasing duration of symptoms. DLB patients whose symptoms had persisted for more than 4 years before their first visit and who had a mean MMSE score of 13.5 showed no statistical difference in tHcy levels compared to those who were symptomatic for less than 2 years and whose mean MMSE score was 17.1 (*P* > 0.05). Alternatively, we found that the frequencies of all four core symptoms were unchanged by illness duration in DLB (*P* > 0.05).

**Table 3:** Clinical and biomedical variables in DLB patients by the duration of cognitive impairment at presentation.

	Clinical variables, Mean ± SD or %						Biochemical variables, Mean ± SD		
Tertile of duration of cognitive impairment, Y	MMSE score	NPI score	CFL	VH	RBD	PARK	tHcy (μmol/L)	Folate (nmol/L)	VitaminB12 (pmol/L)
I<2	17.1 ± 6.9	16.9 ± 16.0	78.90%	68.40%	68.40%	73.00%	31.6 ± 25.5	7.4 ± 3.9	299.2 ± 246.6
II 2–4	15.3 ± 6.8	20.1 ± 17.9	80.90%	76.10%	65.50%	52.30%	21.7 ± 12.8	7.3 ± 6.2	322.3 ± 190.2
III >4	13.5 ± 9.5	21.0 ± 14.7	90.90%	77.20%	54.50%	54.40%	24.0 ± 17.8	9.9 ± 5.0	349.6 ± 213.5
*P* value	0.003	<0.001	0.305	0.553	0.662	0.181	0.196	<0.001	<0.001

*Results are shown as the mean ± the standard deviation for analyses of variance. DLB, dementia with Lewy bodies; MMSE, Mini-Mental State Examination; Hcy, homocysteine; NPI, neuropsychiatric inventory; CFL, cognitive fluctuation; VH, visual hallucination; RBD, rapid eye movement (REM) sleep behavior disorder; PARK, Parkinsonism*.

## Discussion

In this study, we retrospectively observed that elevated levels of plasma tHcy and reduced levels of serum folate were strongly and independently associated with DLB and AD patients. The cumulative frequency plots (Figure 2) showed a stronger association of raised plasma tHcy levels in patients with DLB compared to patients with AD, but the strength of these associations needs further study. Additionally, our study found that the tHcy levels were stable and unaltered by the duration of cognitive impairment before blood sample collection from DLB patients.

The main finding of our case-control study was the robust association of elevated tHcy and reduced folate levels with DLB patients. This study is the first to report a novel positive correlation of tHcy and B vitamin levels with DLB. Results of the previous studies (Luchsinger et al., 2004; Ma et al., 2017) were inconsistent, but the overall results showed that elevated tHcy levels were associated with an increased risk of cognitive impairment, vascular dementia, and AD (Clarke et al., 1991; Seshadri et al., 2002; Ravaglia et al., 2005). The folate and vitamin B12 deficiency might impair methylation reactions to be involved in the pathogenesis of AD (Selhub et al., 1993; Hoffmann et al., 2021). In addition, folate and vitamin B12 are important mediators of tHcy levels, indirectly correlated with AD (Selhub et al., 1993). Accordingly, some (Clarke et al., 1998; Ravaglia et al., 2005; Hoffmann et al., 2021), but not all studies (Ma et al., 2017; Rabensteiner et al., 2020) have shown that low levels of folate and vitamin B12 were associated with AD. More recent studies have specifically addressed the association between a significant elevation of tHcy levels and neurodegenerative diseases including Parkinson’s disease (PD) and multiple system atrophy (MSA; Zhang et al., 2015; Sleeman et al., 2019). To date, the associations of levels of plasma tHcy and serum B vitamins with DLB remain to be established. This prompted us to investigate whether differing tHcy, folate, and vitamin B12 levels may be associated with DLB. This cross-sectional study demonstrated that the DLB group had a higher mean tHcy concentration and a lower mean folate and vitamin B12 concentration compared to the healthy control group. Furthermore, the observed associations of elevated tHcy and decreased folate levels with DLB appeared to be independent of age, sex, education, and known or putative vascular risk factors and were not modified by further adjustments for vitamin B12 and folate or vitamin B12 and tHcy. Conversely, our results suggested that the relationship between the low vitamin B12 levels and DLB was relatively weak and disappeared after simultaneous adjustment for known or putative vascular risk factors. Therefore, there was a strong and independent association of elevated plasma tHcy levels and decreased serum folate levels with DLB, but the association of low vitamin B12 levels was not strong. Notably, impairment in DLB is not restricted to memory as there are broad non-cognitive symptoms such as core features previously related to higher caregiver burden, lower quality of life, and earlier nursing home admission (Galvin et al., 2010; Liu et al., 2017a,b, 2018). Further prospective studies will be required to confirm whether elevated plasma tHcy levels increase the risk of DLB and core clinical features.

Whether the observed association of elevated tHcy levels is a cause or consequence of DLB is a crucial question in need of an answer. Because this was a case-control study, it cannot prove causality. Levels of plasma homocysteine increase with age and are inversely correlated with levels of vitamin B12 and folate in the blood, which are influenced by diet (Selhub et al., 1993). Therefore, it could be argued that DLB resulted in an inadequate dietary intake of folate and vitamin B12 levels, causing an elevation in tHcy levels. Although we cannot refute this possibility, our findings showed that tHcy levels were unaltered by the duration of cognitive impairment in DLB. Patients with DLB whose symptoms had persisted for more than 4 years before their first visit showed no statistical difference in tHcy levels from those who were symptomatic for less than 2 years. A possible explanation is that an elevated tHcy level precedes or appears at an early stage of cognitive impairment in DLB dementia. Next, it is important to determine if an elevated tHcy level plays a possible causative role in DLB in future longitudinal studies.

Consistent with previous studies (Clarke et al., 1991, 1998; Ravaglia et al., 2005; van de Beek et al., 2020), we also found that elevated plasma tHcy levels were strongly associated with AD. We compared the cumulative distribution of tHcy concentrations in the DLB group with those in the AD group, and our results suggested an association between elevated tHcy levels and DLB was stronger than that in AD (Figure 2). The cumulative frequency plots demonstrated a shift in the distribution of the tHcy concentrations to higher values in the DLB and AD group compared with the healthy control group. Furthermore, we identified a significant increase in the tHcy concentrations in the following order: DLB > AD > healthy controls (Figure 2). However, the frequency distribution of the folate and vitamin B12 concentrations in the DLB group was very similar to the distribution observed in the AD group. Therefore, we found that a stronger tHcy association might be observed in patients with DLB compared to patients with AD, but these associations require more studies to confirm their strength. Since most DLB patients had a mix of pathologies—including α-synuclein accumulations as well as AD and cerebrovascular pathology—the stronger association indirectly implied that tHcy might be involved in multiple pathological mechanisms in DLB patients.

Further elucidation of the underlying mechanisms behind the observed associations between elevated tHcy levels and DLB or AD is needed; this could include the following hypotheses. First, homocysteine may exert its neurotoxic effects by activating *N*-methyl-D-aspartate (NMDA) receptors, leading to cell death, or it may be converted into homocysteic acid, an NMDA receptor agonist, which may increase the synthesis of beta-amyloid or have an excitotoxic effect on neurons (Lipton et al., 1997). Second, hyperhomocysteinemia might directly injure endothelial cells causing blood vessel wall damage (Welch and Loscalzo, 1998). Third, hyperhomocysteinemia may contribute to the pathogenesis of PD through an aberrant accumulation of α-synuclein due to the increase of oxidative stress or α-synuclein mRNA (Chao et al., 2000; Macedo et al., 2015). Finally, increasing evidence shows that possible modification of the gut microbiota may be related to AD and PD pathogenesis, promoting immune cell activation and inflammation of the central nervous system (Vogt et al., 2017; Kim et al., 2019). Since DLB and PD are currently considered to be subtypes of an α-synuclein-associated disease spectrum (Lewy body diseases), we theorize that the above mechanisms together may be involved in the pathogenesis of these diseases.

There are several limitations of our study. First, the observed associations in this case-control study, a cross-sectional study, cannot prove causality-our findings must be confirmed in further longitudinal studies. However, our study does include relatively large sample size, a well-defined cohort of Chinese DLB and AD patients, and a clinic-based study design. Second, the core clinical features were rated mainly based on information from medical records and caregivers’ reports, but there were no quantitative data available that measured core features. Third, the serum creatinine concentrations and the status of thyroid function, two major determinants of tHcy levels, were not entered as covariates in the multivariate analysis model in this study. Future studies with further adjustments for the two covariates are needed. Finally, this study included only individuals of Chinese ethnicity and therefore lacks the racial diversity needed for the generalization of the findings.

Our results provide initial evidence that elevated plasma tHcy levels might be independently associated with DLB and AD patients and the association appeared to be stronger for DLB. The stability of tHcy levels over time and the lack of a relationship with symptom duration may refute these observed associations being a consequence of DLB. The possible causative role of tHcy in DLB must be addressed in future longitudinal studies.

## Data Availability Statement

The raw data supporting the conclusions of this article will be made available by the authors, without undue reservation.

## Ethics Statement

The studies involving human participants were reviewed and approved by the medical ethics committee of Capital Medical University, China. The patients/participants provided their written informed consent to participate in this study.

## Author Contributions

GZ, XL, and YJ designed the study, interpreted the data, and drafted and revised the manuscript. SL performed neuropsychological examinations and revised the manuscript. ZC provided statistical analyses and interpreted the results. WH and LM performed experiments and revised the manuscript. ZS and XW drafted and revised the manuscript. GZ wrote the manuscript draft. All authors contributed to the article and approved the submitted version.

## Conflict of Interest

The authors declare that the research was conducted in the absence of any commercial or financial relationships that could be construed as a potential conflict of interest.

## Publisher’s Note

All claims expressed in this article are solely those of the authors and do not necessarily represent those of their affiliated organizations, or those of the publisher, the editors and the reviewers. Any product that may be evaluated in this article, or claim that may be made by its manufacturer, is not guaranteed or endorsed by the publisher.
